# Biological activity versus physiological function of proinsulin C-peptide

**DOI:** 10.1007/s00018-020-03636-2

**Published:** 2020-09-21

**Authors:** Michael Landreh, Hans Jörnvall

**Affiliations:** 1 Departments of Microbiology, Tumor and Cell Biology, Karolinska Institutet, Biomedicum 9C, 171 77 Stockholm, Sweden; 2 Medical Biochemistry and Biophysics, Karolinska Institutet, Biomedicum 9C, 171 77 Stockholm, Sweden

**Keywords:** Protein aggregation, Bioactive peptides, Protein evolution, Diabetes mellitus

## Abstract

Proinsulin C-peptide (C-peptide) has drawn much research attention. Even if the peptide has turned out not to be important in the treatment of diabetes, every phase of C-peptide research has changed our view on insulin and peptide hormone biology. The first phase revealed that peptide hormones can be subject to processing, and that their pro-forms may involve regulatory stages. The second phase revealed the possibility that one prohormone could harbor more than one activity, and that the additional activities should be taken into account in the development of hormone-based therapies. In the third phase, a combined view of the evolutionary patterns in hormone biology allowed an assessment of C-peptide´s role in physiology, and of how biological activities and physiological functions are shaped by evolutionary processes. In addition to this distinction, C-peptide research has produced further advances. For example, C-peptide fragments are successfully administered in immunotherapy of type I diabetes, and plasma C-peptide levels remain a standard for measurement of beta cell activity in patients. Even if the concept of C-peptide as a hormone is presently not supported, some of its bioactivities continue to influence our understanding of evolutionary changes of also other peptides.

## Introduction

Proinsulin C-peptide, or C-peptide, for short, constitutes the mid-section of proinsulin that remains after proteolytic removal of insulin and four basic residues. The human form has 31 residues and is secreted into the blood together with insulin. The discovery of proinsulin by Donald F. Steiner in 1967 contributed to the establishment of the concept of prohormones that require processing to adopt their biologically active forms [1, 2]. It also sparked scientific inquiries into the physiological importance of C-peptide. Today, we know of two such established functions. One is to promote correct folding and disulfide pairing of proinsulin (and hence insulin) during synthesis [3–5], the other to participate in the complex interactions that promote the secretion of insulin from the pancreas [6]. Additional ***activities*** of C-peptide have been sought for since long, and several have been found [7–10], but none has yet been proven to have a true, ***functional*** role.

Two reasons for searches of additional C-peptide activities have been that (1) C-peptide differs from pro-pieces of other peptide hormones in being secreted into the circulation together with the hormone (insulin), and (2) late complications of diabetes are not eliminated (even if delayed) by just insulin treatment alone, hence raising a suspicion of further hormonal deficiency in diabetes [11]. Combined, these two aspects became a stimulus for research on C-peptide, asking if it could be a hormone by itself, and diabetes then a double hormonal deficiency. Gradually, additional activities were found clinically [12–14], extended to molecular observations [15–18], and summarized [10], stimulating still further research. At that time, C-peptide became a “hot” subject, and attracted much attention.

Today, research activity related to the bioactivity of C-peptide has decreased considerably, with significantly fewer in vitro and in vivo studies published than 5–10 years ago. But the decrease does not indicate that C-peptide biology is now fully understood, and a summary now is therefore motivated to clarify the present situation. On the contrary, an additional hormonal activity with biological relevance has still not been established, even if not yet fully excluded at a small scale. Instead, evolutionary explanations now remain [19, 20], and are of interest regarding distinctions between ***activities*** and ***functions*** of peptides in general. Other interest in C-peptide now appears to have faded out [21] when the latest clinical tests also failed to show a clear, hormonal function [22, 23]. So, what has C-peptide research taught us about human physiology, and what possible lessons does it offer for the future? In this report, we outline different lines of enquiry into the role of C-peptide and how they have led to one key question: How can we distinguish between ***biological activity*** and ***physiological function***?

### Three stages of C-peptide research

**Fig. 1:**
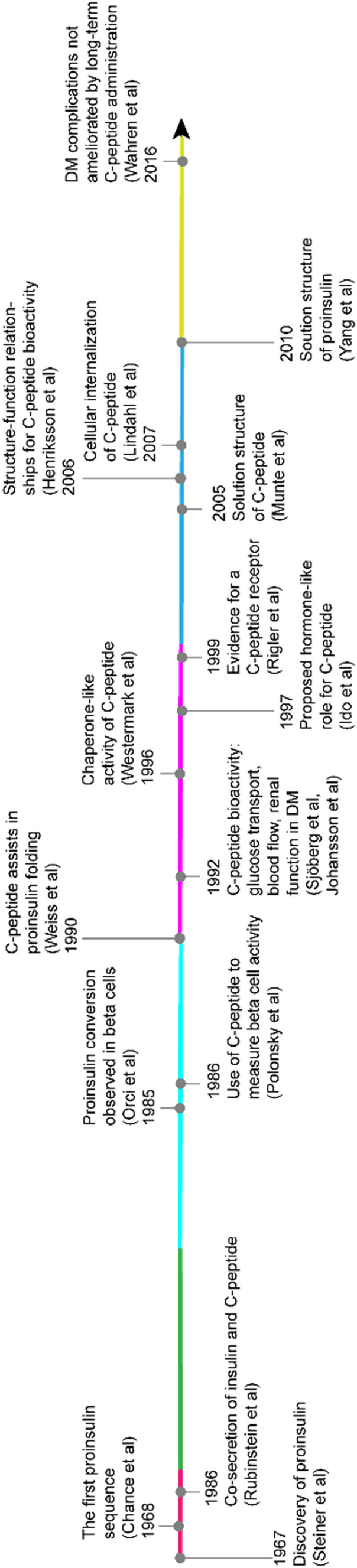
Timeline of the major steps in C-peptide research. Color coding indicates decades between 1967 and 2020

Considering all results hitherto obtained, one can perhaps discern three stages of C-peptide research, even if the phases are intermingled and overlapping (Fig. 1):

- First, a long stage of establishment of the C-peptide pro-form function, its structural variability, and its role in insulin secretion.
- Second, a phase of intense attention, when additional bioactivities were found. At the end of this period, the prospects of a hormone-like C-peptide role and therapeutic use looked promising. Clinical studies and trials were started and continued.
- Third, an evaluation phase, when the biophysical and evolutionary aspects were elucidated. At the end of this period, the sum of C-peptide bioactivities was complex, and non-functional interpretations of C-peptide activities seemed unavoidable.

### Phase 1: discovery of the prohormone and the C-peptide secretion

The phase of C-peptide research from 1967 until approximately 1980 was dominated by two findings related to the discovery of proinsulin:

– There is a pro-form of insulin, discovered by Steiner and co-workers. [1, 2]. This was a surprise at the time but is now viewed as self-evident: biologically, because we now know that peptide hormones generally are synthesized as pro-forms and subsequently cleaved to produce the hormones; and chemically, because the pro-form facilitates the correct folding and disulfide bridge formations.

– Not only the mature hormone, but also C-peptide, as a second part of the pro-form, is secreted into the bloodstream. It was not only the presence of C-peptide in the bloodstream that was an incentive to search for additional activities, but also the higher concentration (nanomolar, versus picomolar for insulin), due to a longer half-life [24, 25]. The latter fact was soon clinically utilized for estimates of insulin production in patients, but it also reinforced questions whether C-peptide might have additional activities. Yet, the prevailing interpretation at the time was that it probably did not. The fact that healthy individuals have a considerable concentration of C-peptide in the blood was instead seen as a sign of little or no function. An observation in retrospect, and a positive lesson for the future might be that if a function is suspected, it should probably be present independent of disease state. In fact, early trials of C-peptide administration in normal and diabetic individuals yielded no significant differences in glucose utilization [26].

**Fig. 2:**
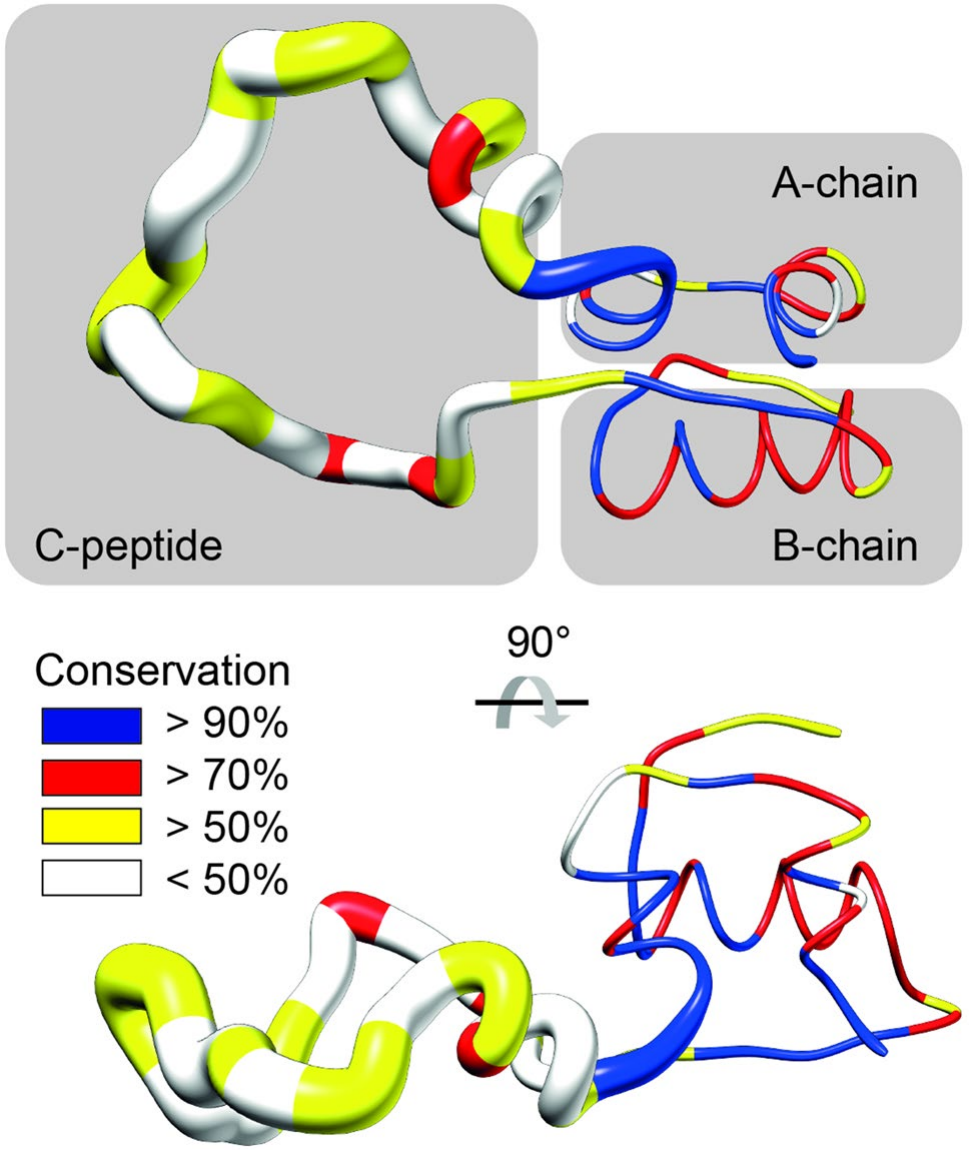
Conformational flexibility and sequence conservation in the C-peptide and insulin parts of proinsulin. Insulin parts of proinsulin, with the NMR structure of proinsulin (PDP ID 2KQP [38]) colored according to conservation [28] and with an increased ribbon thickness indicating a high local flexibility. The NMR structure of proinsulin (PDB ID 2KQP [38]) was colored according to conservation [28]. Increased ribbon thickness indicates high local flexibility as calculated from the NMR structure

It was also discovered that the insulin primary structures varied considerably less than the C-peptide parts between organisms [27, 28], which was early seen as a clue that C-peptide had small chances for a conserved physiological function (Fig. 2). On the other hand, low conservation was soon also noticeable in other peptide hormones, and this feature did therefore not preclude the existence of a physiological function.

By the end of phase one, no specific C-peptide activities except for its role in insulin folding and secretion had been established. Without specific loss of activity in its absence and no absolute structural conservation, in contrast to the corresponding properties of the insulin part, the presence of C-peptide in the blood stream therefore remained an enigma.

### Phase 2: discoveries of multiple hormone-like activities

The picture of C-peptide as an inert byproduct of insulin synthesis changed dramatically with reports on the effects of C-peptide on renal cell function in vitro on cellular preparations [29] and in vivo on rats [14] and in human diabetic patients [30]. Here, C-peptide in vitro stimulated cellular Na^+^/K^+^ ATPase activity and ameliorated renal dysfunctions clinically observable in patients, affecting both subjective and objective clinical signs. As a result, a measurable **bioactivity** had been defined for C-peptide and could now be tested in various settings.

These findings came in a timely fashion for the scientific community: At this stage, the late complications with diabetes were already well known to be delayed by proper insulin regiments, but also not to be ameliorated by insulin alone. The concept therefore remained that C-peptide could be a second hormone, active to combat the development of the late complications with diabetes.

As a result, the molecular mechanisms of the new hormone-like C-peptide activities were soon subject to intense investigations, racing to identify the active site and the target receptor. At the same time, the findings renewed interest in other potential bioactivities, and effects of C-peptide on various cellular processes were soon reported.

To find an active site, C-peptide fragments were employed. It was shown that the C-terminal pentapeptide was sufficient in the assay for Na^+^/K^+^ ATPase activity to give close to full function [15]. Although there was a caveat, that some activity was also observable with other fragments, and even with free glutamic acid, the C-terminal fragment clearly stood out in activity. These findings turned out to converge with efforts to find a C-peptide receptor. Indeed, specific binding of C-peptide to membrane preparations [17, 31] and subsequently, also binding of just the C-terminal pentapeptide to the same membranes [32], was demonstrated. The method used was novel, involving fluorescence-correlation spectroscopy, and was remarkable, since traditional radio-isotope binding assays had previously not detected any specific binding. However, the novel method revealed both an actual binding and a low binding constant, compatible with normal C-peptide serum concentration levels and with membrane binding sites more or less fully occupied already in healthy individuals. Thus, all seemed to agree with a hormonal concept of C-peptide, its binding to cellular membrane receptors and a saturation difference in healthy *versus* diabetic patients. Suddenly, the hunt for possible C-peptide receptors and C-peptide active fragments appeared to have borne fruit.

However, there were caveats here, too: In rat models, C-peptide effects could be elicited with peptides that had inverted chirality or sequence [14]. For the membrane binding assays, calculations suggested that the previously used radio-label assays should also have been able to detect receptor binding at this nanomolar level, but they did not [17]. However, these investigations still supported a hormone-like role for C-peptide with an active site at the C-terminal pentapeptide [33].

While effects of C-peptide on renal function where consolidated, additional bioactivities emerged, showing that liberation of cellular calcium signals could be coupled to C-peptide in calcium-release assays [18]. These findings again indicated specific receptor binding by the five C-terminal residues of C-peptide, and for specific cellular responses by calcium ion signals. Similarly, C-peptide was shown to influence MAP-kinase activities [34, 35]. Additionally, further clinical tests suggested specific C-peptide effects in local blood circulation tests, on the speed of nerve conductivity in diabetic patents and on C-peptide effects on QT variations in EKG measurements of diabetic patients [21]. At this stage, a review on all bioactivities of C-peptide was published in Diabetologia [10], together with a counter-opinion review [36], and the general opinion was quite positive on the hormonal concept of C-peptide and its possible role in late diabetic complications.

### Phase 3: combined knowledge of structure, function and evolution of C-peptide distinguishes bioactivities from functional roles

Driven by the possibility to understand C-peptide bioactivities, investigations were launched into the structural features of C-peptide, both as a part of proinsulin and as a free peptide in solution. Computational studies suggested a flexible architecture, potentially with a turn motif around residues 15–20 [37]. Solution NMR then provided the first high-resolution structure, confirming that C-peptide lacks a well-defined fold but exhibits some helical propensity in the C-terminal pentapeptide (Fig. 2) [38]. Overall, the structural studies consistently found three important features: The acidic residues at the N-terminus, the flexible middle segment capable of adopting a turn-like structure, and the relatively conserved overall C-peptide length of approximately 30 residues [39].

Additionally, C- peptide was found to be able to self-associate [40]. This finding was surprising given that the sequence contains multiple glutamic acid residues, resulting in high solubility and significant electrostatic repulsion. However, electrophoresis and immunoblotting, as well as non-denaturing mass spectrometry revealed the presence of dimers, trimers, tetramers, and even larger oligomers at physiological C-peptide concentrations. Metal ions were found to reduce self-association, whereas low pH and the presence of the cationic detergent SDS led to an increase, suggesting that charge interactions play a crucial role in the association process. Oligomers formed in vitro could be visualized via Thioflavin T fluorescence, indicating the presence of amyloid-like states [41]. NMR studies showed that low pH shifted the conformational landscape of C-peptide towards a higher β-strand content. Electron microscopy, X-ray diffraction and FT-IR eventually confirmed that C-peptide, despite being highly soluble at physiological pH, spontaneously can form amyloid-like fibrils under acidic conditions [41, 42].

This behavior remarkably parallels that of insulin, which interacts with metal ions and forms large oligomers at low pH that eventually assemble into fibrils [43, 44]. Proinsulin with intact linkages between B-, C-, and A-chains, on the other hand, is much less prone to aggregate, which implies a possible relationship between the stabilities of C-peptide and insulin [45]. As shown by mass spectrometry, C-peptide was found to reduce non-specific insulin oligomerization as a first step in aggregation [18, 46]. Prolonged incubation of both peptides under amyloid-forming conditions delayed fibril formation and eventually resulted in shorter fibrils and amorphous aggregates [47]. The pH-dependence of the insulin/C-peptide interaction was found to stem from charge-based contacts between glutamic acid residues in C-peptide and basic side-chains in the insulin B- and A-chains, leading to mutual charge neutralization and reduced solubility in the 2:1 insulin-C-peptide complex [48]. Consequently, changes in pH caused the dissociation of the complex, releasing soluble peptides. These findings led to the conclusion that C-peptide is adapted to promote solubility of the peptide hormones stored at high concentrations in the pancreatic beta cells [49]. Hence, the role of C-peptide in secretion seems compatible with the molecular studies. In line with this conclusion, co-administration of insulin with C-peptide in diabetic patients promoted the uptake of insulin, which may be due to increased bioavailability driven by disaggregating effects of C-peptide [18]. However, all in vitro observations did not fully correlate with the situation found in vivo. Although small C-peptide-rich deposits were detected in vessels of diabetic patients, these deposits did not exhibit amyloid-like features [50].

By this stage, the wide spread of potential C-peptide bioactivities raised questions of how such diverse functions, all of which would require molecular interactions, could be accommodated in such a short peptide as C-peptide and with so little sequence conservation. Generally, receptor activation relies on sequence motifs that co-evolve with their receptor counterparts, as shown for the insulin receptor itself [51]. In contrast, the combined information from multiple C-peptide fragment studies indicated that the different bioactivities are associated with different peptide segments [39]. As a result, local conservations were concluded to reflect the biological relevance of each segment’s bioactivity, and local interactions in the C-peptide evolution may be compatible with its overall role and low conservation.

A more detailed picture can be obtained by insights form structural studies and their relationship with activities of C-peptide. Remarkably, we then see that all of the conserved biophysical features relate to the folding and stability of insulin [52]. The N-terminal glutamic acid residues, which share a co-evolution pattern with several residues on the A- and B-chains, help proinsulin folding. Their negative charge counter-balances the positive net charge of insulin, shifting the isoelectric point away from the acidic conditions found inside the beta cell granules. Due to its length and flexibility, C-peptide protrudes from the folded proinsulin, which likely facilitates its efficient excision during processing [53].

Importantly, the suggestion that the only consistent characteristics of C-peptide relate to its insulin partner could imply that the other features of the C-peptide sequence are not subject to restrictions, and that their associated bioactivities have not been consolidated by the evolutionary pressure [52]. This observation may also shed light on a central point that has been raised repeatedly in connection with C-peptide biology: No C-peptide mutation has been discovered that could be reliably connected to a clinical phenotype. Similarly, comparisons of Diabetes types I and II have not revealed any differences attributable to a lack of C-peptide, although only type I can be considered a “double-deficiency” disease. While it cannot be excluded at this stage that some complications can be connected to the absence of C-peptide, even in-depth genetic studies have not yielded causative relationships between C-peptide mutations and any hereditary diabetes-related condition [54]. In 2016, a large-scale clinical study finally failed to detect any significant effects of C-peptide administration in type I diabetic patients [23].

### Is C-peptide redundant?

At the end of the third phase in C-peptide research, a broad range of bioactivities had been established, but also a lack of deficiency phenotype, a lack of evolutionary conserved features, and a lack of clinical effects in randomized controlled trials (Table 1). Combined, this led to the conclusion that C-peptide is not essential to the major metabolic processes, and that its most direct role in physiology is likely limited to its originally proposed function in the production and storage of insulin. These conclusions ran counter to the possibilities for hormonal actions of C-peptide. Yet C-peptide undoubtedly had a number of molecular and cellular activities. Therefore, at the end of stage three, a question seems obvious: Is C-peptide redundant?

**Table 1:** Summary of C-peptide biological activities that lack the corresponding hallmarks of physiological function. See text for details and further references

Property	Evidence for C-peptide biological activity	Lack of evidence for corresponding C-peptide physiological function
Diabetic complications	- Complications not ameliorated by insulin administration alone [11]	- No phenotype identified for C-peptide mutations [54]
Presence	- A C-peptide segment present in proinsulins across all phyla [20]	- C-peptide segments are poorly conserved [27]
Requirements	- C-peptide required for correct folding of proinsulin and prevents insulin aggregation in vitro [45, 52]	- C-peptide mutations do not impair insulin biogenesis [54]
Interactions	- C-peptide binds to cell membranes and activates MAP kinases [16, 17, 31, 34]	- A C-peptide receptor not conclusively identified [56]
Neurological effects	- C-peptide reduces neuropathy and influences nerve conductivity in individual DMT1 patients [12]	- No beneficial effects on nerve function observed in large-scale clinical trials [23]
Cellular effects	- C-peptide elicits a broad range of cellular effects in tissue cultures and rodent models [10]	- I*n vitro* C-peptide effects exhibit only limited specificity for peptide sequence or chirality [14]

The most likely answer to the apparent contradictions is that most of the reported biological activities, if not all, are dispensable, because of rare occurrence in vivo, or are easily compensated for by other peptides. Yet, several of the activities are robust and have been demonstrated repeatedly. Finally, the existence of C-peptide across all *vertebrates* as a connecting peptide in proinsulin still suggests a surprising resilience to evolutionary elimination, and is in contrast to its sequence variability.

### Distinctions between biological activities and physiological functions

As we have seen, the presence of a connecting peptide is a conserved feature of proinsulin. Whatever the exact role of this peptide may be, it places little evolutionary pressure on the rest of C-peptide, thus leaving much of the other properties open to variation. This unique scenario, a peptide that is constantly expressed but remains free to evolve provides an evolutionary space for genesis of new **biological activities**, which may eventually become **physiological functions**. The inconsistent antagonistic activities that C-peptide displays in vitro and in vivo may then be the result of natural sequence variations, but remain subject to change as long as they have not become consolidated. This could change if a randomly generated bioactivity amounts to an evolutionary advantage, or if a compensatory mechanism is lost elsewhere. Under this regime, bioactivities such as the disputed activation of the orphan GPCR 146 by C-peptide [55, 56] and the related cross-interactions with insulin signaling, could represent a nascent physiological function for C-peptide in its fine-tuning of insulin activity, but remain difficult to trace until they are associated with a specific phenotype. It is therefore not inconceivable that C-peptide functions may already have emerged on a small scale, as in specific cell types, but have not yet been diversified to further cell types. In this limited sense, the view of C-peptide as a bioactive peptide may be correct, without disproving the lack of essential hormonal functions.

- Today, we may ask whether the present conclusions about a non-physiological importance of C-peptide bioactivities could have been reached earlier. However, as indicated above, each step towards understanding the role of C-peptide has followed from new experimental possibilities, coordinating results from many types of biochemical, biophysical, computational and clinical research. Hence, it is difficult to discern a possible “short-cut” to the present conclusions, other than research itself. Thus, the progress appears all the time to have been further research, exactly like the stages above illustrate. Yet three rules can be extracted from the C-peptide investigations:
- Evolutionary properties are strong indicators on the extent of functional importance.
- Lack of clinical deficiencies in diseased patients are also strong indications on the non-functional importance regarding molecular observations.
- Lack of additional symptoms in healthy individuals upon overdoses forms a third strong indication on a lack of functional importance.
- Each of the three "warnings" can be overlooked by multiple peptide actions and then be misinterpreted regarding functional meaning. But as a lesson for future peptide research, one or two, but perhaps all three warnings should not have been overlooked that long in the interpretations of functional conclusions for C-peptide.
